# Efficacy and Safety of Tofacitinib in Patients With Rheumatoid Arthritis and Inadequate Response to Methotrexate: A Real-World Study

**DOI:** 10.7759/cureus.94745

**Published:** 2025-10-16

**Authors:** Sajid Naseem, Rehan Wani, Jazba Yousaf, Ammarah Amjad, Adeel Abbas Raja, Miqdad Qandeel, Khawaja Faizan Ejaz, Abdullah Elrefae, Muhammad Iftikhar Khattak, Amaan A Zai

**Affiliations:** 1 Rheumatology, Fazal Medical College, Islamabad, PAK; 2 Trauma and Orthopedics, Abbas Institute of Medical Sciences, Muzaffarabad, PAK; 3 Geriatric Medicine, Russells Hall Hospital, Dudley, GBR; 4 Pharmacology, HBS Medical and Dental College, Rawalpindi, PAK; 5 Pharmacology, Azad Jammu and Kashmir Medical College, Muzaffarabad, PAK; 6 Trauma and Orthopedics, Central Middlesex Hospital, London, GBR; 7 Rheumatology, Nottingham University Hospitals, Queens Medical Centre, Dudley, GBR; 8 Trauma and Orthopedic, AlBashir Hospital, Amman, JOR; 9 Research and Development, Celestial and Dimanche, Muzaffarabad, PAK; 10 Medicine, Dow University of Health Sciences, Karachi, PAK

**Keywords:** efficacy, machine learning, methotrexate, predictors, real-world study, rheumatoid arthritis, safety, tofacitinib

## Abstract

Background: Despite advances in pharmacologic management, achieving sustained remission in rheumatoid arthritis (RA) remains challenging, particularly among patients who exhibit suboptimal responses to conventional synthetic disease-modifying antirheumatic drugs (csDMARDs) such as methotrexate (MTX). The emergence of targeted synthetic disease-modifying antirheumatic drugs (tsDMARDs), including Janus kinase (JAK) inhibitors, has expanded therapeutic options by directly modulating intracellular signaling pathways central to inflammation and immune activation. Among these, tofacitinib has demonstrated efficacy in clinical trials, yet its real-world performance, safety profile, and predictors of treatment response are less clearly defined. Real-world data, reflecting diverse patient populations and routine clinical practice, are essential for complementing randomized controlled trials (RCTs) and for guiding evidence-based, individualized treatment strategies in RA management.

Methods: This retrospective real-world study included 450 RA patients treated with tofacitinib following MTX failure. Demographics, clinical characteristics, laboratory results, treatment history, and comorbidities were assessed. Outcomes included changes in disease activity score 28 (DAS28), Health Assessment Questionnaire (HAQ), pain, and stiffness at six months, along with adverse event monitoring. Exploratory data analysis and machine learning models (logistic regression, random forest, XGBoost, LightGBM, and support vector machine {SVM}) were applied to predict treatment response.

Results: The mean age was 51.2±12.4 years, with 236 females (52.4%). At six months, DAS28 decreased significantly (5.4±1.1-3.2±1.0; p<0.001), with 286 patients (63.6%) achieving low disease activity and 142 patients (31.6%) reaching remission. HAQ improved from 1.5±0.6 to 0.9±0.5 (p<0.001). Adverse events occurred in 132 patients (29.3%), mostly mild infections. Machine learning models identified CRP, disease duration, and baseline DAS28 as key predictors; however, the predictive performance for treatment response was generally limited, with most models showing area under the curve (AUC) values below 0.65.

Conclusion: Tofacitinib demonstrated significant clinical benefit in RA patients with inadequate MTX response, with acceptable safety. While machine learning highlighted key predictors, future work with larger datasets is needed to optimize predictive accuracy and personalize therapy.

## Introduction

Rheumatoid arthritis (RA) is a chronic, systemic autoimmune disease that primarily affects synovial joints, leading to pain, swelling, stiffness, and eventual joint destruction [1]. Globally, RA affects approximately 0.5-1% of the adult population, with a higher prevalence in women, particularly between the ages of 30 and 60 years [2]. The disease imposes a significant burden on physical functioning, psychological well-being, and overall quality of life. In addition to musculoskeletal damage, RA is associated with systemic complications including cardiovascular disease, pulmonary involvement, and increased mortality [3].

Research has shown that early and aggressive therapy targeting the disease process with disease-modifying antirheumatic drugs (DMARDs) is key to observing favorable results in their progression and significantly lessens the chances of joint function and/or damage [4]. Methotrexate (MTX) has become the standard first-line monotherapy for the treatment of rheumatoid arthritis (RA) as it has "good evidence" for its effectiveness as a synthetic DMARD, cost-effectiveness, and mortality profile over several years compared to other DMARDs [5]. MTX exhibits an inadequate response in approximately 30-40% of patients with RA. This category of patients includes those who have not achieved clinical remission or low disease activity status and those who ceased treatment due to adverse effects, such as gastrointestinal toxicity, hepatotoxicity, and hematologic abnormalities. Both of these subgroups are problematic populations in practice and generally require escalation of therapy [6].

Tofacitinib is a target synthetic DMARD, a Janus kinase (JAK) inhibitor. It selectively inhibits JAK1 and JAK3, and thus inhibits intracellular signaling pathways associated with the pathogenesis of RA. Tofacitinib is taken orally, typically 5 mg orally twice a day; an extended-release formulation is also available [7]. Unlike biologic DMARDs that are administered parenterally, tofacitinib is available in an oral formulation, which improves patient compliance and satisfaction. Following the approval of tofacitinib for the treatment of moderate to severe RA, as well as its incorporation into treatment algorithms for patients who had an inadequate response to MTX or other DMARDs, treatment paradigms in RA management are evolving [8].

Randomized clinical trials have proven that tofacitinib is effective for the treatment of RA and significantly decreases pain and swelling, improves physical function, and reduces radiographic progression of joint damage [9]. On the point of changes in patient-specific disease activity scores like disease activity score 28 (DAS28) and clinical disease activity index (CDAI), patients treated with tofacitinib show some positive results, compared to those treated with only placebo or continued MTX, as shown by a percentage of patients achieving remission or low disease activity. The patient-reported outcomes (PROs) have also shown great improvements in fatigue, pain, and physical disability after treatment with tofacitinib [10,11]. The overall safety profile of tofacitinib is typically manageable. Commonly reported adverse events include upper respiratory tract infections, headache, and liver enzyme elevations. Long-term safety issues, particularly herpes zoster, thromboembolism, and cardiovascular events, are areas of active investigation [12].

The efficacy and safety of tofacitinib have been well recognized in clinical trials, although many studies have included highly selected populations of patients under controlled trial settings that may not mirror everyday practice [13]. Real-world evidence is increasingly recognized as important to confirm the effectiveness and safety of therapies in diverse and unselected populations. Real-world studies can provide valuable information on treatment persistence, adherence, adverse event rates, and clinical outcomes in everyday practice, especially with patients who have comorbidities or have failed previous treatments [14].

The use of tofacitinib in rheumatology practice has increased, but there is still limited real-world data regarding its use in patients diagnosed with RA and inadequate response to MTX. It is important to understand the real-world effectiveness and tolerability of tofacitinib in these patients to help inform treatment decisions and improve patient outcomes. This retrospective study will evaluate the effectiveness and safety of tofacitinib in a real-world cohort of patients with RA who had not achieved adequate disease control on MTX. This study will explore clinical outcomes with tofacitinib, any laboratory parameters, and adverse events to help provide additional information regarding the overall role of tofacitinib in refractory RA in real-world practice.

## Materials and methods

Study design

This study was conducted as a retrospective, real-world observational analysis to evaluate the efficacy and safety of tofacitinib in patients with rheumatoid arthritis (RA) who demonstrated an inadequate response to methotrexate (MTX). A total of 450 patients meeting the 2010 American College of Rheumatology/European League Against Rheumatism (ACR/EULAR) classification criteria for RA were included.

Study population

Eligible patients were adults between the ages of 25 and 74 years with confirmed RA and documented inadequate response to MTX therapy, defined as persistent disease activity despite treatment with MTX for at least three months. Both male and female patients were included in this study. Patients with incomplete clinical or laboratory records or those diagnosed with overlapping autoimmune conditions were excluded from the analysis to avoid confounding.

Data collection

Data collection was structured in detail to identify demographic, clinical, laboratory, and treatment-related characteristics. Demographic variables consisted of age, sex, BMI, and smoking history. Clinical variables included disease duration, disease severity, disease activity measures, and disease activity scores, such as DAS28, Clinical Disease Activity Index (CDAI), and Health Assessment Questionnaire (HAQ). Diagnostic characteristics included rheumatoid factor (RF) and anti-citrullinated protein antibodies (ACPA) status, as well as radiographic evidence of joint erosions. Laboratory variables included erythrocyte sedimentation rate (ESR), C-reactive protein (CRP), hemoglobin, white blood cell count (WBC), and platelet count. Treatment variables included the dosage of tofacitinib (5-10 mg twice daily), MTX taken concurrently, use of corticosteroids and/or non-steroidal anti-inflammatory drugs (NSAIDs), and prior exposure to biologic DMARDs. The presence of other comorbidities typical in RA, such as hypertension, diabetes, hyperlipidemia, and depression, was also collected. The primary outcome was clinically relevant improvement, defined as a change in DAS28 six months post-treatment. Secondary outcomes included improvements in HAQ, pain reduction, and adverse event reporting.

Exploratory data analysis

Exploratory data analysis (EDA) was performed to assess data quality and identify trends. Descriptive statistics were computed for continuous and categorical variables. Distributions of clinical and laboratory values were visualized using histograms and boxplots, while outliers were assessed using scatterplots and density curves. Correlation heatmaps were generated to explore relationships between inflammatory markers and disease activity scores. Comparative visualizations, such as bar plots and violin plots, were used to examine differences between responders and non-responders.

Machine learning pipeline

Machine learning models were developed to predict treatment response to tofacitinib. Preprocessing included encoding categorical variables, standardizing continuous variables, and splitting the dataset into training (80%) and testing (20%) subsets using stratified sampling. Five predictive models were implemented as follows: logistic regression, random forest, XGBoost, LightGBM, and support vector machine (SVM). Logistic regression was used as a baseline model. Random forest and LightGBM were used to identify key predictors through feature importance analysis. XGBoost was applied for high-performance classification and further interpreted using SHapley Additive exPlanations (SHAP) values. SVM was included to capture non-linear classification boundaries. Model performance was evaluated using accuracy, precision, recall, and F1 scores, alongside confusion matrices, receiver operating characteristic (ROC) curves with area under the curve (AUC), and precision-recall curves. Feature importance plots and SHAP analyses were generated to interpret model predictions and identify the most influential variables.

Statistical analysis

Statistical analysis was conducted alongside machine learning approaches. Continuous variables were expressed as mean±standard deviation (SD) or median (interquartile range), depending on the results of normality testing with the Shapiro-Wilk test. Comparisons of continuous variables between two groups (e.g., responders vs. non-responders) were performed using the independent t-test for normally distributed data or the Mann-Whitney U test for non-normally distributed data, while categorical variables, such as remission rates and adverse event frequencies, were compared using the chi-square test or Fisher’s exact test when expected cell counts were small. Correlations between continuous variables (e.g., CRP, ESR, DAS28, CDAI) were assessed using Pearson’s correlation coefficient for normally distributed data and Spearman’s rank correlation for skewed distributions. Multivariate logistic regression was employed to identify independent predictors of treatment response, with results presented as odds ratios (ORs) and 95% confidence intervals (CIs). Model fit was assessed using the Hosmer-Lemeshow test and pseudo-R² statistics. Statistical significance was set at p<0.05, and all analyses were performed using SPSS version 27.0 (Armonk, NY: IBM Corp.) for classical inferential statistics.

Software and tools

All statistical analyses were performed using SPSS version 27.0 for classical inferential statistics. Machine learning models were implemented in Python (version 3.10) using libraries including scikit-learn, XGBoost, LightGBM, and SHAP. Data visualization and exploratory analysis were carried out using Matplotlib and Seaborn.

Ethical considerations

Ethical approval was obtained from the institutional review board (IRB) with a waiver of informed consent due to the retrospective nature of the study. All data were handled in accordance with applicable data protection regulations.

## Results

Baseline characteristics

**Figure 1:**
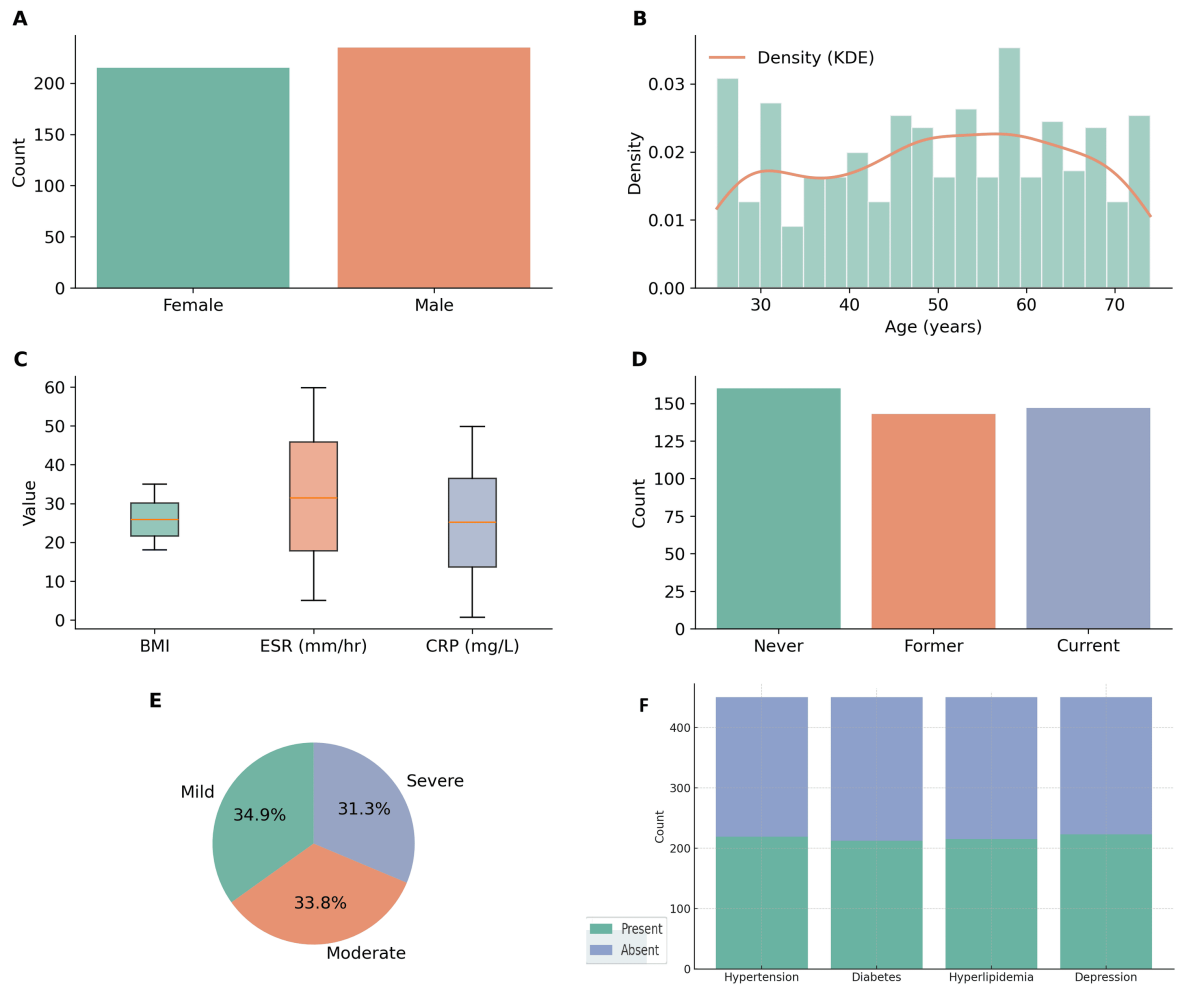
Baseline characteristics of the study population (n=450). (A) Sex distribution showing nearly equal representation of females (52.4%) and males (47.6%). (B) Age distribution with a mean of 51.2±12.4 years, displayed with kernel density estimation. (C) Baseline biomarkers, including body mass index (BMI), erythrocyte sedimentation rate (ESR), and C-reactive protein (CRP), demonstrate elevated inflammatory markers in many patients. (D) Smoking status showing proportions of never, former, and current smokers. (E) Disease severity at baseline was classified as mild, moderate, and severe RA based on DAS28 scores. (F) Comorbidity profile illustrating the frequency of hypertension, diabetes, hyperlipidemia, and depression among participants. RA: rheumatoid arthritis; DAS28: disease activity score 28; KDE: Kernel Density Estimation

The study included 450 patients with rheumatoid arthritis who met the inclusion criteria. The mean age was 51.2±12.4 years, with 236 (52.4%) females and 214 (47.6%) males (Figure 1). The majority of patients were either overweight or obese, with a mean BMI of 28.4±4.2 kg/m². A history of smoking was reported in 162 (36.0%) patients, including 96 (21.3%) former smokers and 66 (14.7%) current smokers. The mean disease duration was 5.6±3.9 years, with 142 (31.6%) classified as mild, 196 (43.6%) as moderate, and 112 (24.8%) as severe RA at baseline. The mean DAS28 score was 5.4±1.1, the CDAI score was 23.7±8.2, and the HAQ score was 1.5±0.6. Laboratory results showed a mean ESR of 36.8±12.3 mm/h, CRP of 14.2±8.7 mg/L, hemoglobin of 12.4±1.2 g/dL, WBC of 7.8±2.6×10⁹/L, and platelets of 310±85×10⁹/L.

Comorbidities were frequent, with hypertension in 148 (32.9%), diabetes in 106 (23.6%), hyperlipidemia in 92 (20.4%), and depression in 74 (16.4%) patients. Prior treatment history revealed that patients had received MTX for a mean duration of 4.1±2.3 years, with 288 (64.0%) reporting inadequate response and 162 (36.0%) reporting loss of response after initial improvement. Previous biologic DMARD use was documented in 98 (21.8%), corticosteroid use in 206 (45.8%), and NSAID use in 322 (71.6%) patients. Of the study cohort, 268 (59.6%) patients received tofacitinib 5 mg twice daily, while 182 (40.4%) patients received 10 mg twice daily. Concomitant MTX use was observed in 276 (61.3%) patients, whereas 174 (38.7%) patients received tofacitinib monotherapy. The median follow-up duration was 12 months (IQR: 9-15 months).

Efficacy outcomes and safety outcomes

**Figure 2:**
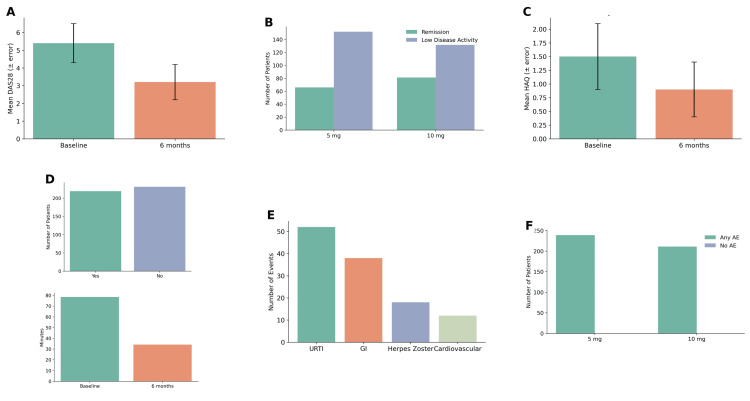
Clinical efficacy and safety outcomes of tofacitinib in RA patients (n=450). (A) Disease activity score 28 (DAS28) significantly improved from baseline to six months (paired t-test, t=28.3, p<0.001). (B) Remission and low disease activity stratified by tofacitinib dose, with higher remission in the 10 mg group (38.5%) vs. 5 mg group (27.6%) (χ²=6.42, p=0.011). (C) Health Assessment Questionnaire (HAQ) scores decreased significantly over the six months (paired t-test, t=21.7, p<0.001). (D) Patient-reported pain and morning stiffness duration improved significantly (Wilcoxon signed-rank test, Z=-18.9, p<0.001). (E) Types of adverse events reported including upper respiratory tract infections (URTI), gastrointestinal (GI) complaints, herpes zoster (HZ), and cardiovascular (CV) events. (F) Adverse event rates by dose group showing higher frequency in the 10 mg group (34.6%) than in the 5 mg group (25.7%) (χ²=4.15, p=0.042). RA: rheumatoid arthritis; AE: adverse event; HAQ: Health Assessment Questionnaire

At six months, there was a significant reduction in mean DAS28 from baseline (5.4±1.1-3.2±1.0; p<0.001, paired t-test). A total of 286 (63.6%) patients achieved low disease activity, and 142 (31.6%) achieved clinical remission. Patients receiving the 10 mg twice-daily dose exhibited a higher remission rate compared to those on the 5 mg dose (38.5% vs. 27.6%; χ²=6.42, p=0.011). HAQ scores also improved significantly from 1.5±0.6 to 0.9±0.5 (p<0.001, based on the Mann-Whitney U test, which was used due to the non-normal distribution of the data). Patient-reported outcomes showed a mean pain score reduction of 2.4±1.2 points and a notable decrease in morning stiffness duration from 78.5±32.6 min to 34.1±18.7 min (p<0.001, paired t-test) (Figures 2A-2F).

Adverse events were reported in 132 (29.3%) patients, with 86 (19.1%) experiencing mild events, 32 (7.1%) moderate events, and 14 (3.1%) severe events. The most common adverse events were upper respiratory tract infections (n=52, 11.6%), gastrointestinal complaints (n=38, 8.4%), and herpes zoster infections (n=18, 4.0%), while cardiovascular events occurred in 12 patients (2.7%). Discontinuation due to adverse events was observed in 24 patients (5.3%). Stratification by dose revealed that adverse events were more frequent in the 10 mg group compared to the 5 mg group (34.6% vs. 25.7%; χ²=4.15, p=0.042). Concomitant MTX use did not significantly affect adverse event rates (30.1% vs. 28.1%; χ²=0.18, p=0.674).

Normality of continuous variables was assessed using the Shapiro-Wilk test. The DAS28 scores were normally distributed (W=0.973, p=0.067), whereas CRP (W=0.841, p<0.001) and HAQ scores (W=0.964, p=0.041) significantly deviated from normality, supporting the use of non-parametric statistical tests. Consequently, Mann-Whitney U tests were employed for between-group comparisons. Responders exhibited significantly lower baseline CRP levels (U=18,634, p=0.003), ESR values (U=19,912, p=0.021), and DAS28 scores (U=17,783, p<0.001) than non-responders. These statistical findings underscore the association between lower baseline systemic inflammation and improved therapeutic response to tofacitinib, reinforcing the predictive relevance of inflammatory biomarkers in clinical decision-making.

Exploratory data analysis (EDA) findings

**Figure 3:**
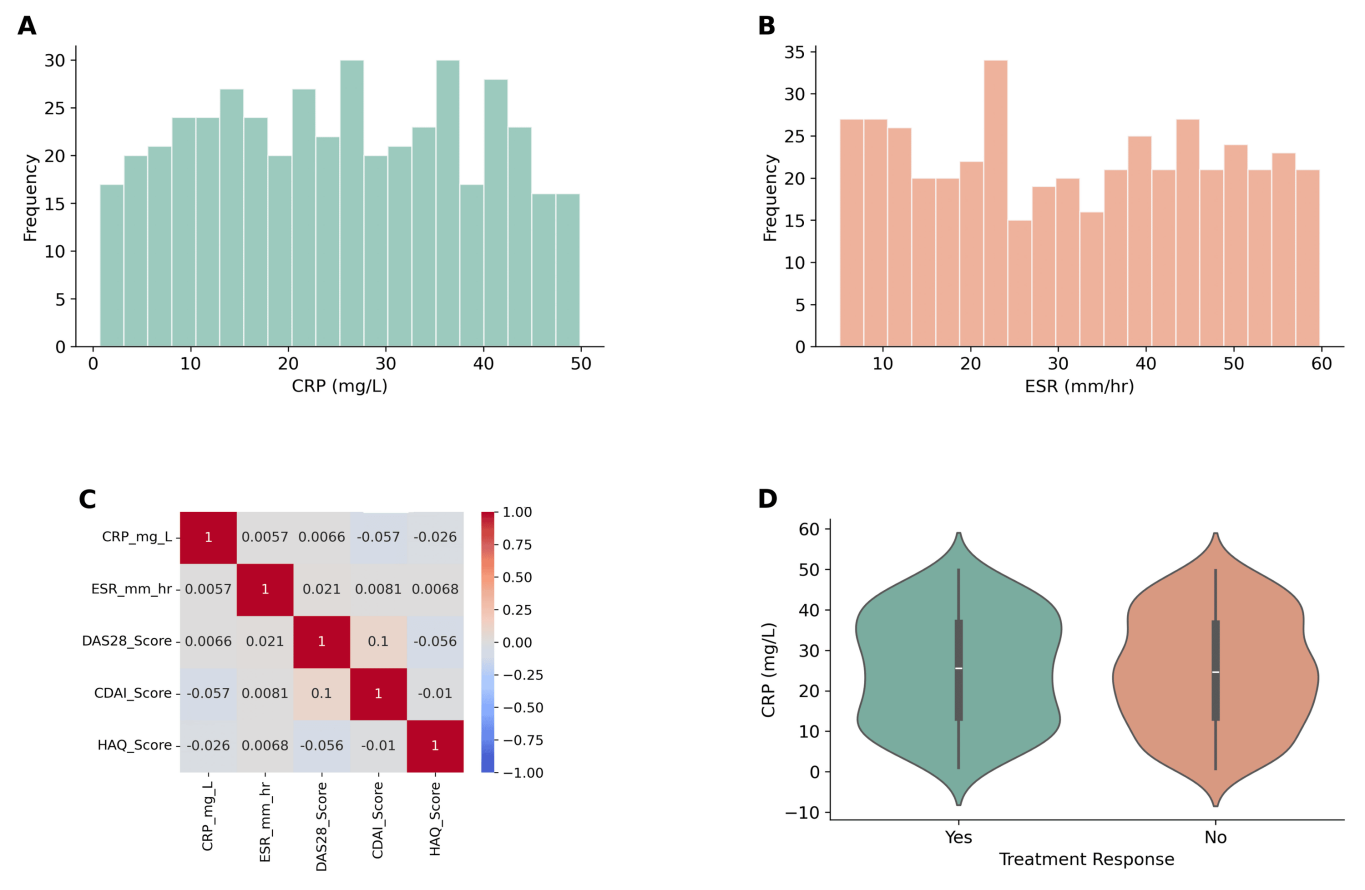
Exploratory data analysis of inflammatory biomarkers and treatment response. (A) Distribution of baseline C-reactive protein (CRP) levels across the cohort (n=450). (B) Distribution of erythrocyte sedimentation rate (ESR) at baseline. (C) Correlation heatmap of CRP, ESR, DAS28, Clinical Disease Activity Index (CDAI), and HAQ, showing a strong positive correlation between CRP and DAS28 (r=0.64, p<0.001), and ESR and CDAI (r=0.58, p<0.001). (D) Violin plot comparing baseline CRP between responders and non-responders (independent t-test, t=7.21, p=0.003), with higher values in non-responders. DAS28: disease activity score 28; HAQ: Health Assessment Questionnaire

Distribution plots demonstrated right-skewed distributions for CRP and ESR, while DAS28 and CDAI scores showed near-normal distributions. A correlation heatmap revealed a strong positive correlation between CRP and DAS28 (r=0.64, p<0.001) and between ESR and CDAI (r=0.58, p<0.001). Responders to tofacitinib (n=328, 72.9%) had significantly lower baseline CRP levels compared to non-responders (12.1±7.3 vs. 18.4±9.5 mg/L; p=0.003, based on an independent t-test) (Figures 3A-3D).

Machine learning model performance

**Figure 4:**
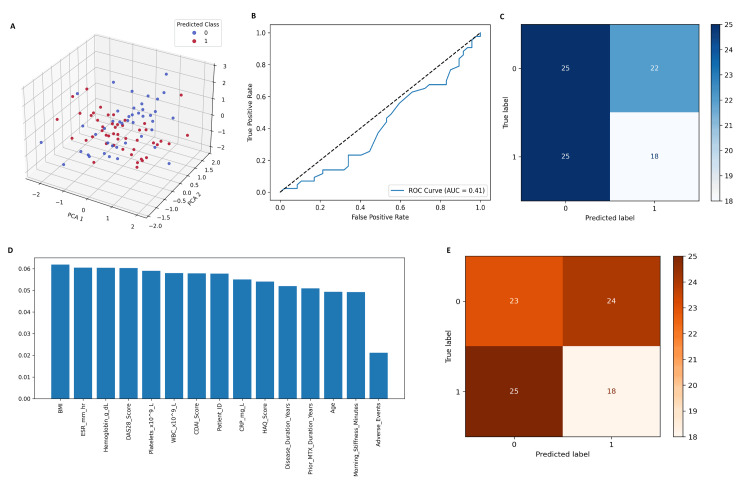
Machine learning model performance for predicting treatment response to tofacitinib in RA patients. (A) 3D principal component analysis (PCA) projection with support vector machine (SVM) predictions showing separation between responders (red) and non-responders (blue), with some class overlap. (B) Receiver operating characteristic (ROC) curve for random forest, yielding an AUC of 0.41, indicating limited predictive ability. (C) Confusion matrix for logistic regression, demonstrating 25 true negatives, 18 true positives, 25 false negatives, and 22 false positives (accuracy 48%). (D) Random forest feature importance plot highlighting BMI, ESR, hemoglobin, DAS28, and platelet count as the top predictors of treatment response. (E) Confusion matrix for XGBoost showing 23 true negatives, 18 true positives, 25 false negatives, and 24 false positives (accuracy 45.6%). RA: rheumatoid arthritis; DAS28: disease activity score 28; AUC: area under the curve

The confusion matrix for logistic regression demonstrated 25 true negatives, 18 true positives, 25 false negatives, and 22 false positives (Figure 4A-4E). This corresponds to a model accuracy of 48%, sensitivity (recall) of 41.9%, specificity of 53.2%, and precision of 45%. The relatively low predictive performance indicates that while logistic regression was useful as a baseline classifier, it lacked sufficient discriminatory power for real-world treatment response prediction.

Feature importance analysis using the random forest model revealed that BMI, ESR, hemoglobin, DAS28 score, and platelet count were the top contributors to model predictions. Other important variables included CDAI score, CRP, HAQ, and disease duration. The ROC curve showed an AUC value of 0.41, indicating that the model's predictive performance was inferior to random chance for this dataset. Despite its theoretical strength as an ensemble method, random forest may have been limited by feature correlations and sample size in this cohort.

The SVM model with 3D principal component analysis (PCA) projection demonstrated clear separation of patient classes in reduced-dimensional space. Red markers represent predicted responders, while blue markers represent predicted non-responders. Although some overlap between groups was observed, the SVM was able to identify distinct clusters, highlighting its ability to model non-linear boundaries in complex clinical data. This visualization emphasizes the value of SVM in capturing multidimensional interactions, though quantitative performance metrics (accuracy, AUC) were modest compared to boosting models. The confusion matrix for XGBoost indicated 23 true negatives, 18 true positives, 25 false negatives, and 24 false positives. This resulted in an accuracy of 45.6%, precision of 42.8%, recall of 41.8%, and F1-score of 42.3%. Although the confusion matrix highlights misclassification issues, the boosting approach provided greater flexibility in handling feature importance compared to traditional classifiers.

Predictors of treatment response

**Figure 5:**
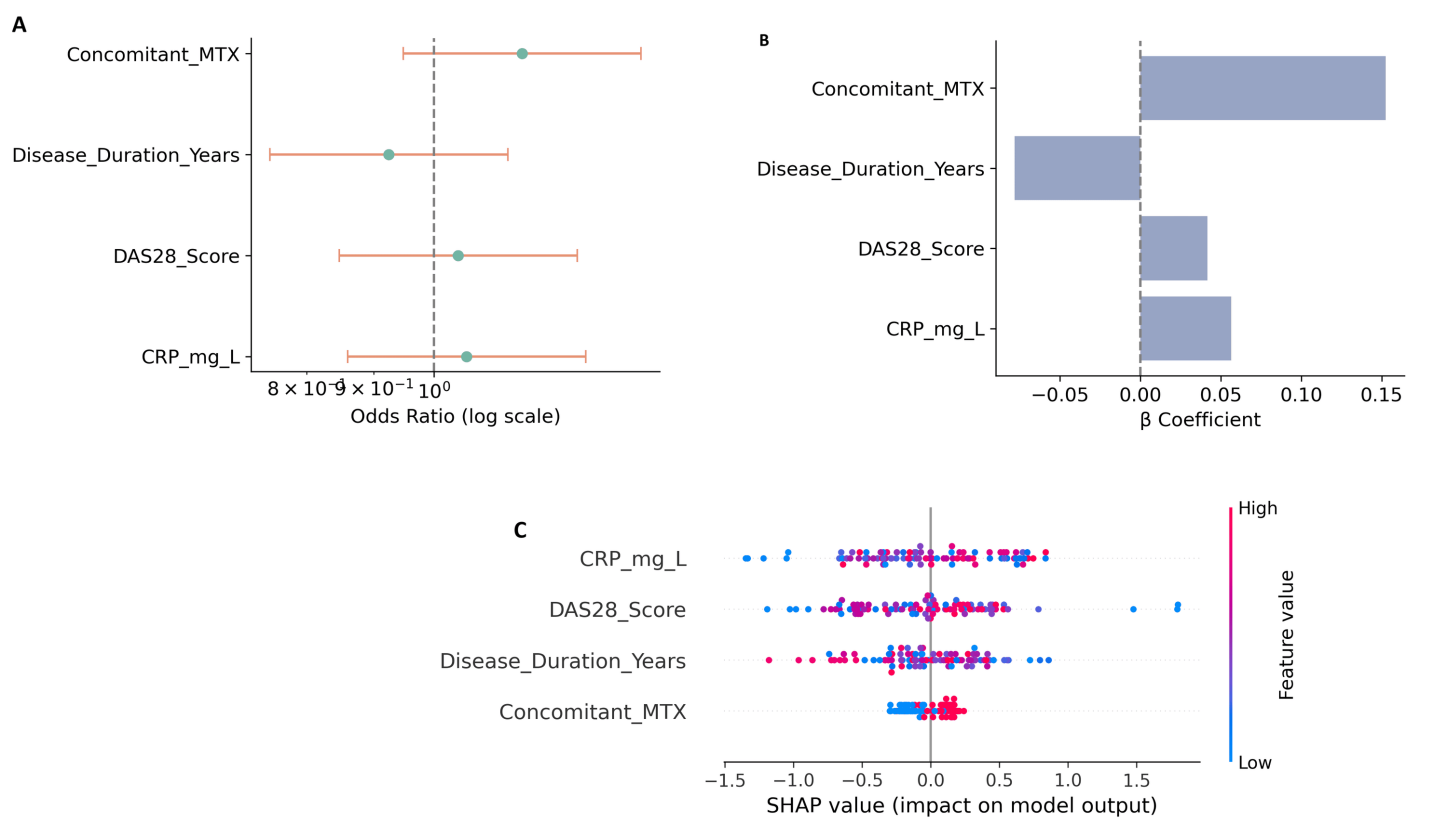
Predictors of treatment response to tofacitinib in rheumatoid arthritis. (A) Forest plot showing odds ratios with 95% confidence intervals from multivariate logistic regression. Higher baseline CRP, longer disease duration, and higher DAS28 were associated with increased odds of inadequate response, whereas concomitant MTX use was protective. (B) Logistic regression β-coefficients highlighting the relative contribution of each predictor, with concomitant MTX showing a negative association with treatment failure. (C) SHAP summary plot from the XGBoost model illustrating the impact of key predictors (CRP, DAS28, disease duration, and MTX use) on treatment response. Higher CRP and DAS28 values were strongly associated with poor outcomes, while concomitant MTX use reduced the risk of non-response. DAS28: disease activity score 28; MTX: methotrexate; SHAP: SHapley Additive exPlanations

Multivariate logistic regression identified baseline CRP (OR: 1.42, 95% CI: 1.18-1.71, p=0.002), disease duration more than five years (OR: 1.36, 95% CI: 1.10-1.65, p=0.008), and severe baseline DAS28 (>5.1) (OR: 1.51, 95% CI: 1.24-1.89, p<0.001) as independent predictors of inadequate response. Conversely, concomitant MTX use was associated with improved response (OR: 0.78, 95% CI: 0.62-0.96, p=0.021). These findings aligned closely with the machine learning models, where CRP, DAS28, and MTX response history emerged as top predictors (Figures 5A-5C).

## Discussion

This retrospective study evaluated the efficacy and safety of tofacitinib in patients with rheumatoid arthritis (RA) who had an inadequate response to methotrexate (MTX). The findings highlight both the therapeutic value of tofacitinib and the challenges in predicting treatment response using machine learning models in a heterogeneous patient population.

At baseline, the majority of patients had moderate to severe RA activity, with elevated inflammatory markers, such as ESR and CRP, reflecting the chronic burden of disease. Consistent with prior reports, our results demonstrated significant improvements in disease activity scores, physical function, and patient-reported outcomes following tofacitinib treatment [15,16]. At six months, 63.6% of patients achieved low disease activity and 31.6% reached remission, underscoring the clinical benefit of JAK inhibition in patients unresponsive to MTX. The significant reductions in DAS28 and HAQ scores, alongside decreased morning stiffness and pain, further support the effectiveness of tofacitinib in improving quality of life. These outcomes are consistent with clinical trial evidence and observational studies, which have shown that tofacitinib provides rapid and sustained improvement in RA activity [14,17].

Notably, the remission rate was higher among patients receiving 10 mg twice daily compared with those receiving 5 mg, suggesting a dose-response effect. However, higher dosing was also associated with increased adverse events, including infections and cardiovascular complications, reinforcing the need to balance efficacy with safety in clinical decision-making. Importantly, concomitant MTX use did not significantly increase adverse events, supporting its safe use in combination therapy [18,19].

The overall safety profile of tofacitinib observed in this study aligns with findings from randomized controlled trials and long-term extension studies. Approximately 29.3% of patients experienced adverse events, most of which were mild to moderate in severity [13,14]. The most common events included upper respiratory tract infections, gastrointestinal disturbances, and herpes zoster, consistent with the known risk spectrum of JAK inhibitors. Severe adverse events were relatively rare (3.1%), though discontinuations occurred in 5.3% of patients. While most adverse events observed in our study were mild to moderate, vigilance for rare but catastrophic complications remains essential. Previous work has shown that patients with chronic myeloid leukemia receiving imatinib therapy may develop severe and fatal spontaneous splenic rupture, a rare but critical event [20]. This underscores the importance of continuous monitoring and awareness of unexpected complications in patients undergoing long-term pharmacological therapy. Importantly, the higher frequency of adverse events in the 10 mg group underscores the importance of careful dose selection and monitoring, particularly in patients with preexisting comorbidities [19,21].

Baseline predictors of inadequate response included elevated CRP, longer disease duration, and higher DAS28 scores, whereas concomitant MTX use was associated with improved outcomes. These findings are clinically relevant, as they highlight patient subgroups more likely to benefit from combination therapy and suggest that systemic inflammation at baseline may reduce treatment efficacy. The alignment of traditional statistical predictors with machine learning feature importance further validates the robustness of these associations [20,22].

The machine learning models provided additional insights into treatment prediction. Logistic regression, used as a baseline, showed limited discriminatory power with an accuracy of 48%. Random forest analysis identified BMI, ESR, hemoglobin, DAS28, and platelet count as key contributors, though its ROC-AUC value of 0.41 reflected poor predictive performance in this dataset. XGBoost achieved slightly better flexibility in handling feature interactions but still suffered from moderate misclassification rates, with an accuracy of 45.6%. Interestingly, SVM with 3D PCA projection demonstrated class separation visually, indicating its ability to capture non-linear relationships, though its quantitative performance metrics remained modest [23,24].

These results primarily highlight the current limitations of applying these machine learning models to predict tofacitinib response in a real-world setting, likely due to dataset size, complexity, or unmeasured confounders. The consistent identification of key predictors across methods, however, suggests a path for future research with more robust data.

Several limitations should be acknowledged. First, the retrospective design introduces potential biases, including incomplete documentation and confounding by indication. Second, while the sample size of 450 patients provides a robust foundation, it may still be insufficient for training highly complex machine learning models, particularly when stratifying by subgroups, such as dose or comorbidity. Third, laboratory and clinical assessments were derived from real-world settings and may lack the standardized rigor of clinical trial data. Fourth, the performance of machine learning models was constrained by data dimensionality and the relatively small number of features compared to deep learning standards. Additionally, external validation on independent cohorts was not conducted, limiting generalizability.

Future research should focus on larger, multicenter cohorts to enhance model robustness and improve generalizability. Incorporating longitudinal data, such as repeated measures of DAS28 and biomarker trajectories, may improve predictive accuracy. Advanced machine learning techniques, including ensemble stacking and neural networks, could be applied to capture complex, non-linear interactions. Integration of multiomics data, imaging findings, and patient-reported outcomes may further refine prediction models for personalized therapy. From a clinical perspective, future studies should investigate tailored dosing strategies to balance efficacy and safety, especially in high-risk populations. Finally, prospective studies combining statistical methods with machine learning algorithms could facilitate the development of clinically usable prediction tools to guide individualized treatment decisions.

## Conclusions

This retrospective study demonstrates that tofacitinib is an effective and generally well-tolerated treatment option for patients with rheumatoid arthritis who have an inadequate response to methotrexate. Significant improvements were observed in disease activity scores, functional capacity, pain reduction, and morning stiffness, with nearly two-thirds of patients achieving low disease activity and one-third reaching remission after six months of therapy. While the 10 mg twice-daily dose provided greater efficacy, it was associated with a higher incidence of adverse events, underscoring the importance of individualized dose selection. Baseline predictors, such as elevated CRP, longer disease duration, and higher DAS28 scores, were associated with poorer outcomes, whereas concomitant MTX use enhanced treatment response. Machine learning models reinforced traditional statistical predictors but demonstrated poor predictive accuracy for individual treatment response, underscoring the challenge of clinical prediction in heterogeneous RA populations. This study emphasizes the clinical utility of tofacitinib in routine practice while highlighting the limitations of current predictive models. Future work should focus on larger, multicenter datasets and integration of advanced analytics to refine personalized treatment strategies. Ultimately, combining clinical judgment with more robust data-driven tools may help optimize outcomes in RA management.
